# Clinical experience with modified Miccoli’s endoscopic thyroidectomy for treatment of thyroid carcinoma in 86 cases

**DOI:** 10.1186/2047-783X-18-51

**Published:** 2013-12-01

**Authors:** Xiang Shen, Zhi-ming Miao, Wei Lu, Da-li Gu, Dan Yang, Hao Shen, Feng Geng

**Affiliations:** 1 Department of Breast Surgery, the First People Hospital of Zhangjiagang, No 68 Jiyang West Road, Zhangjiagang, Jiangsu 215600, China

**Keywords:** Thyroid carcinoma, Endoscopy, Lymph node dissection, Modified Miccoli’s, Thyroidectomy

## Abstract

**Background:**

The main purpose of this study was to assess the feasibility and relevant applying techniques of total thyroidectomy for thyroid carcinoma with a modified Miccoli’s approach.

**Methods:**

Eighty-six patients with thyroid carcinoma consecutively received radical operation from October 2007 to June 2012. A cavity above the gland was constructed by a modified suspension method using the suspension retractor with suction catheter after the pathway making. Eighty-six cases underwent the modified Miccoli’s endoscopic thyroidectomy using the ultrasonic scalpel and the space maintain-regulating device. Level VI lymph node dissection was performed using the method of inspection pit.

**Results:**

All the procedures were completed successfully. The average detection rate of level VI lymph nodes, the average time of thyroidectomy and lymph nodes dissection were 7.27 ± 3.99 pieces per case, 51.32 ± 13.35 min, and 38.43 ± 15.24 min, respectively. With regard to postoperative complications, there were three cases of delayed transient hoarseness, two patients with transient numbness of hands and feet, one subject with chylous fistula, and no hemorrhage.

**Conclusion:**

Total thyroidectomy for thyroid carcinoma can be safely performed with the modified Miccoli’s approach by using ultrasonic scalpel and the space maintain-regulating device. Application of these adaptive reforms can obviously reduce the difficulties in manipulation and have the advantages of minimal incisions, good cosmetic results, less bleeding, shorter hospital stay, and fewer complications.

## Background

Thyroid carcinoma is rare among human malignancies, with a reported incidence of < 1%, but is the most frequent endocrine cancer, accounting for around 5% of thyroid nodules [1]. The mainstay treatment of thyroid carcinoma is surgical resection. Traditionally, Miccoli is a minimally-invasive video-assisted thyroid surgery combining endoscope and open view [2,3]. However, the disadvantages of the traditional Miccoli’s surgery were obvious, such as single incision, small operational space, and instability of cavity caused by manual draw-off. A special mechanical suspension device (a regulator to build cavity) was invented by Professor Gao Li from Sir Run Run Shaw hospital in Zhejiang province, China [4]. It allows a practical modified Miccoli procedure with the improvement on the technology of cavity building. Indeed, the modified Miccoli’s surgery derived from endoscopic thyroidectomy can be applied in total thyroidectomy for thyroid carcinoma including: lobectomy for tumor side, isthmusectomy, subtotal lobectomy for another side, and the clearance of VI region lymph nodes for tumor side. The aim of this work was to evaluate the feasibility and application of this new technique in clinical practice.

## Methods

Records of 86 patients with a diagnosis of a pure papillary thyroid carcinoma, registered with the Department of Breast Surgery at the First People Hospital of Zhangjiagang, and treated with a modified Miccoli’s approach between October 2007 and June 2012, were reviewed. The characteristics of patients are illustrated Table 1, according to the medical history. The age of participants was in the range of 22 to 68 years (mean age, 46.92 ± 10.78 years; 73 women, 13 men). Sixty-seven cases were diagnosed with micro focus carcinoma via the intraoperative rapid pathological sections. There were 26 cases of multi-focus, 60 cases of single-focus, 10 cases of bilateral carcinoma, and 76 cases of unilateral carcinoma observed. The size of the tumors was in the range of 0.2 to 3.0 cm, with the average diameter of 0.95 ± 0.69 cm.

**Table 1:** Detailed baseline of the patients

**Characteristic**		***n*** **= 86 (%)**
Sex	Male	13 (15%)
	Female	73 (85%)
Age (mean ± SD, years)	46.92 ± 10.78	
Lateral type	Bilateral carcinoma	10 (8.6%)
	Unilateral carcinoma	76 (88.4%)
Papillary tumor stage	I	35 (40.5%)
	II	40 (46.5%)
	Not stated	11 (13%)
Micro-focus or not	Yes	67 (77.9%)
	No	19 (22.1%)
Tumor size (mean ± SD, cm)	0.95 ± 0.69	
	Single	26 (30.2%)
Lesion location	Multiple	60 (69.8%)
	Not stated	0

### Surgical technique

**Figure 1:**
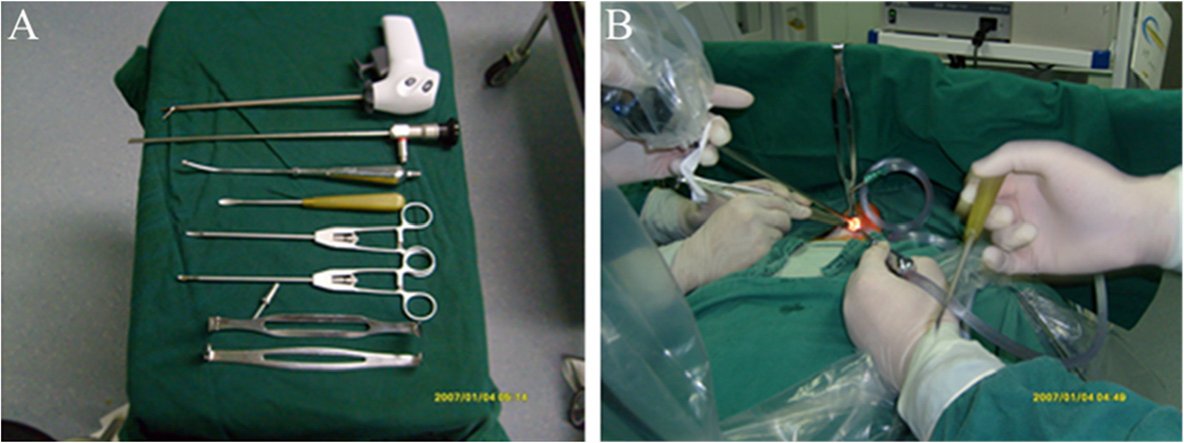
**Surgical instruments and the surgical scene of the modified Miccoli’s endoscopic thyroidectomy. (A)** Surgical instruments; **(B)** surgical scene.

All procedure was approved by the ethic committee of the First People Hospital of Zhangjiagang. Patients were fixed on the operating table in the supine position at general anesthesia. A single transverse incision of 2 cm was made in the middle area of the neck, approximately 2 cm above the sternal notch. The epithelial and subdermal flap on the surface of ribbon muscles were then separated about 1 cm distance. A 2–2.5 cm linea alba cervicalis incision reached the true-pseudocapsule gap on the front of thyroid isthmus, separating the strap muscles from the underlying thyroid. The retractor with suction catheter was placed on the gap of ribbon muscles from the cephalic of the incision. The other side of the retractor was suspended on the cross bar (Figure 1).

**Figure 2:**
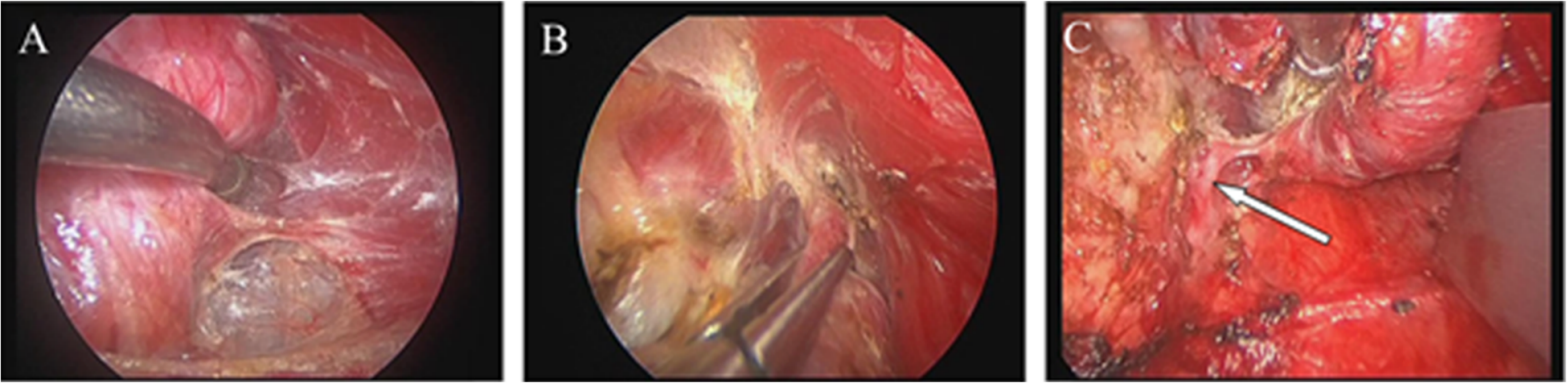
**The key operative procedure of thyroidectomy. (A)** Ready to amputate the middle thyroid vein; **(B)** the treatment on thyroid upper blood vessel; **(C)** the treatment on the entrance point of thyroid recurrent laryngeal nerve.

**Figure 3:**
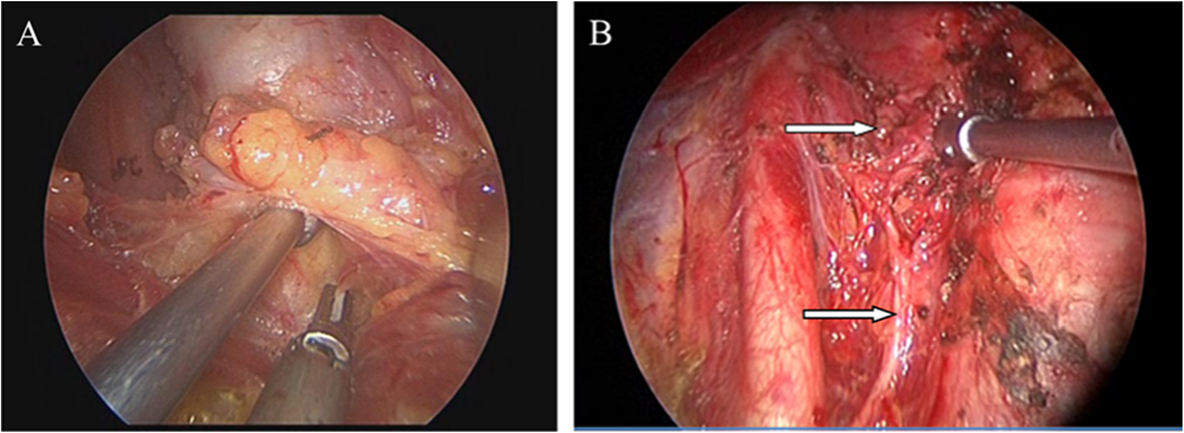
**The key operative procedure of clearance of the level VI lymph nodes. (A)** The clearance of lymph nodes in the central area before the trachea was isolated; **(B)** the recurrent laryngeal nerve and superior parathyroid gland after the clearance of lymph nodes in the central area.

During the operation, the endoscope of 5 mm and 30 degrees was held up by the first assistant, and the lateral retractor was tensioned by the second assistant. The bending ultrasonically activated scalpel was the major suction dissector combined with the blunt stripper and other instruments. The lacuna was expanded until the thyroid and lateral margin were completely exposed under the endoscope vision. The optical magnification allowed an excellent vision of both the external branch of the superior laryngeal nerve and the recurrent nerve, which were prepared together with the upper parathyroid gland. The vessels were ligated between clips or with the scalpel until the lobe, completely freed, could be extracted by gently pulling it out through the skin incision. The priority step performed was lesions resection, followed by total thyroidectomy, subtotal thyroidectomy, and clearance of the level VI lymph nodes. The incision was closed by means of two subcuticular stitches and a skin sealant. Drainage was necessary (Figures 2 and 3).

### Postoperative management

The incision routine placed on the negative pressure ball (patent number 201020664616.6) for thyroid. Patients fasted on the day of the operation. Postoperative oral feeding was resumed the next day, and the decannulation took place after 2 or 3 days. All patients were discharged 48 h after surgery to better evaluate the postoperative outcomes. Direct laryngoscopy and ultrasonography were used after the 1-week postoperative follow-up.

## Results

Eighty-six cases were treated with modified Miccoli’s but not with open surgery. Clearance of the level VI lymph nodes was used in all 86 cases including 29 total resection and 57 partial resection. The average length of incision was 2 cm, the mean time of thyroidectomy was 51.32 ± 13.35 min; the average time of lymph nodes dissection was 38.43 ± 15.24 min; the intraoperative blood loss was 0 to 30 mL, with an average of 5 mL; the average hospital stay was 2.79 ± 1.03 days. A total of 626 pieces of level VI lymph nodes were detected, and the average detection rate of the level VI lymph nodes was 7.28 ± 3.99 pieces per case. Eighty-three pieces of metastatic lymph nodes were included and 29 cases of patients with node-positive, accounting for 33.72% of the total.

**Figure 4:**
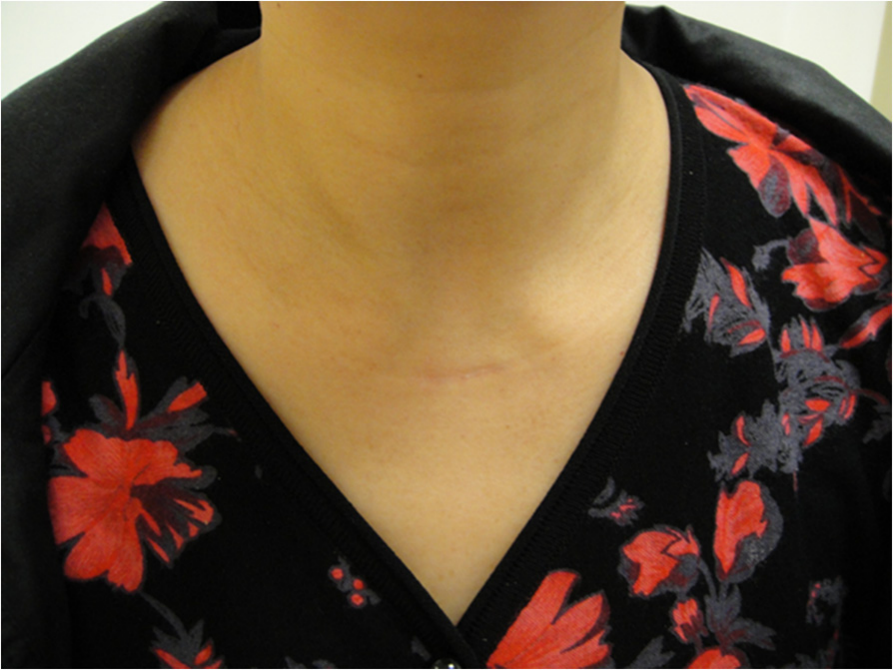
The cosmetic result after the modified Miccoli’s endoscopic thyroidectomy.

Postoperative complications included: (1) three cases of delayed transient hoarseness appeared on the second day: vocal cord edema was found under the laryngoscope (they all recovered 1.5 months after surgery); (2) two cases of transient numbness of hands and feet appeared after surgery: these symptoms disappeared after calcium supplementation for about 1 week; and (3) one case of chylous fistula appeared after surgery and recovered in the third week by extubation. Mild edema was observed in postoperative incision, which disappeared in about 1 month and led to an excellent cosmetic result (Figure 4). All the patients evaluated the overall operation as very satisfactory or satisfactory 2 weeks after surgery.

## Discussion

At present the thoroughness and safety of primary tumor resection and clearance of the level VI lymph nodes have become the controversial focus of endoscopic thyroidectomy. Multi-focus lesion is an important characteristic of differentiated thyroid carcinoma. In order to remove the cancer completely, the total thyroidectomy or subtotal thyroidectomy is usually performed assisted by postoperative iodine-131 radiotherapy [5]. However, considering that the growth rate of differentiated thyroid carcinoma is slow and the patients have a long survival phase, some scholars advocate to use relatively conservative surgeries to the low-risk papillary carcinoma patients (aged < 45 years, tumor diameter < 2 cm, no capsule invasion, and with negative cervical lymph node), including subtotal thyroidectomy, ipsilateral lobectomy, and isthmectomy. These surgeries could reduce complications such as recurrent laryngeal nerve and parathyroid injuries. Subtotal thyroidectomy has become a regular surgical style in China. Lymph node metastasis is common in the central (level VI) lymph node, and repeated operation could increase the risk of recurrent laryngeal nerve and parathyroid injury. Clearance of the level VI lymph nodes is the conventional choice whether the neck lymph nodes swell or not after clinical check [6].

Level VI contains the thyroid gland, and the adjacent nodes bordered superiorly by the hyoid bone, inferiorly by the innominate (brachiocephalic) artery, and laterally on each side by the carotid sheaths [7]. Lymphatic flow received by level VI lymph nodes is mainly from thyroid, laryngeal, and hypopharynx. This group generally includes four to 12 lymph nodes. The scope of clearance of the level VI lymph node is from the outside to the inside of the carotid artery [8].

The traditional Miccoli is similar to traditional open surgery because of the different characteristics of endoscopic thyroid surgery operations. The intuitively exposed operative field makes it more suitable for thyroid resection [9-17]. However, it is difficult for traditional Miccoli to apply thyroidectomy and clear the level VI lymph nodes because of the narrowness and instability of the cavity and lack of special surgical instruments. In this study, we improved special surgical instruments and operation technology: (1) the cavity was built by a simple suspension method using suspension retractor with suction catheter to keep the airiness of cavity and a clear vision. The surgery process would not be influenced by changing bend suction catheter into other instruments during the operation; (2) the handle length of the suspension retractor was shortened appropriately, and the height of the ultrasonic scalpel and suspension device was separated to avoid mutual interference; (3) more flexible surgical vision was available by increasing the angle of endoscopic from 30 degrees to 70 degrees; (4) it was convenient for precise anatomy of tissues by choosing 23 cm length of tiny orthogonal separation clamp; and (5) lateral retractor with supported endoscope was used to increase the stability of endoscope.

There are several points for attention in modified Miccoli’s endoscopic thyroidectomy procedures: (1) middle thyroid veins were cut by ultrasonically activated scalpels when we dealt with the lateral margin. The recurrent laryngeal nerve near the throat was then bluntly separated, and gland blood vessels were ligated or cut. At the same time, we paid attention to protect the parathyroid and made sure no residual fat tissue samples were on the surface of the thyroid; (2) thyroid gorge and suspensory ligament of thyroid gland were cut to increase the flexibility of thyroid. Attention must be paid to protect the tracheal wall and recurrent laryngeal nerve; (3) to deal with upper blood vessels of thyroid, the gap around the thyroid must be separated by the nerve dissector. The outside of the gap and cervical vagina vasorum were separated, but the upper blood vessels did not need to be separated alone to reduce the possibility of bleeding. The anterior and gorge branch of upper blood vessels were first cut along with the thyroid, and then pulled down the upper blood vessel still along the thyroid. To ensure the blood supply of parathyroid, we kept a rear adipose tissue as far as possible and a certain length of vascular; (4) the branch on the surface of thyroid was cut after being separated. The capsule of thyroid was isolated by the nerve stripper and only the branch of entrance into thyroid was cut. In order to protect the parallel fiber tissues, the recurrent laryngeal nerve from the inferior thyroid artery and lateral face of cricoid cartilage may be exposed; and (5) after the suspensory ligament was completely cut off, we used nerve stripper to isolate the recurrent laryngeal nerve under the condition of protecting the parathyroid. Ultrasonic scalpel was taken outward by 2 mm when we cut the thyroid on the surface of the recurrent laryngeal nerve.

The advantages of clearance of the level VI lymph nodes under modified Miccoli’s endoscopic thyroidectomy are as follows: (1) the amplifying view is helpful for the operators’ precise surgical procedures. It can also clear level VI lymph nodes more thoroughly and protect the important tissues including parathyroid, recurrent laryngeal nerve, and superior laryngeal nerve better; (2) as the incision is located above the sternal incisure, it is better to reveal posterior notacoria gap of thyroid, esophageal groove on the front, and bilateral trachea; (3) this technique could shorten the operation time, reduce operation damage, and speed up postoperative recovery because no extension of the incision is needed. These features indicate this surgery is minimally invasive and therefore can achieve attractive cosmetic results (Figure 4); (4) if neck lymph node clearance is needed, it could reduce the damage of important tissues, including recurrent laryngeal nerve, parathyroid, and so on.

Several points for attention in clearance of the level VI lymph nodes under modified Miccoli’s endoscopic thyroidectomy are as follows: (1) the incision should be as low as possible but above the sterna notch to expose the region of trachea under endoscope, and the length of incision should be about 1.0 cm; (2) the suspension retractor should be appropriately shorten to avoid mutual interference with the ultrasonic scalpel; (3) when the right side is cleansed, separated along the vessels of brachiocephalic trunk to carotid artery surface, it should not be deviated to the outside to prevent damage to the right vagus; (4) the upper boundary of the affected side should not be over the cricoid cartilage, and care should be taken over the parathyroid on the affected side; (5) fiber fatty connective tissues of the contralateral trachea were cut into thin layers and exposed to protect the two side of recurrent laryngeal nerve; (6) the contralateral upper bound should not be over the lower pole of the thyroid; (7) care should be taken to distinguish esophageal muscles to prevent damage to the esophageal wall when the fiber fatty connective tissues of the two sides of recurrent laryngeal nerve were cleansed; and (8) recurrent laryngeal nerve of the affected side should be exposed during the operation. When using the ultrasonic scalpel, the function bar of ultrasound scalpel should not contact any nerve to prevent damage; the distance between the function bar and nerve should be at least 2 to 3 cm.

## Conclusions

In conclusion, with the improvement of surgical instruments and operation technique, the modified Miccoli’s endoscopic thyroidectomy has been proven safe and feasible. By this surgical technique, we have obtained excellent results with patient cure rate and comfort, with shorter hospital stay, less postoperative pain, and more attractive cosmetic results. We believe that modified Miccoli’s endoscopic thyroidectomy is worth popularizing.

### Consent

Written informed consent was obtained from the patient for the publication of this report and any accompanying images.

## Competing interests

The authors declare that they have no competing interests.

## Authors’ contributions

XS and ZM M participated in the design of this study, and they both performed the statistical analysis. WL carried out the study, together with DLG, DY collected important background information, and drafted the manuscript. HS and FG conceived of this study, and participated in the design and helped to draft the manuscript. All authors read and approved the final manuscript.
